# In-person versus remote (mHealth) delivery for a responsive parenting intervention in rural Kenya: A cluster randomized controlled trial

**DOI:** 10.21203/rs.3.rs-4733054/v1

**Published:** 2024-08-16

**Authors:** Italo Lopez Garcia, Jill Luoto, Frances Aboud, Pamela Jervis, Teresa Mwoma, Edith Alu, Aloyce Odhiambo

## Abstract

Background An estimated 43% of children under age 5 in low- and middle-income countries (LMICs) experience compromised development due to poverty, poor nutrition, and inadequate psychosocial stimulation. Numerous early childhood development (ECD) parenting interventions have been shown to be effective at improving ECD outcomes, at least in the short-term, but they are a) still too expensive to implement at scale in low-resource and rural settings, and b) their early impacts tend to fade over time. New approaches to deliver effective ECD parenting interventions that are low-cost, scalable, and sustainable are sorely needed. Methods Our study will experimentally test a traditional in-person group-based delivery model for an evidence-based ECD parenting intervention against a hybrid-delivery model that increasingly substitutes in-person meetings for a remote (mHealth) delivery via smartphones, featuring audiovisual content and WhatsApp social interactions and learning. We will assess the relative effectiveness and cost of this hybrid-delivery model against purely in-person delivery and will extend the interventions over two years to increase their ability to sustain changes in parenting behaviors and ECD outcomes longer-term. Our evaluation design is a cluster Randomized Controlled Trial (cRCT) across 90 villages and approximately 1200 households. Midline and endline surveys collected 12 and 24 months after the start of the interventions, respectively, will examine short- and sustained two-year intention-to-treat impacts on primary outcomes. We will also examine the mediating pathways using Mediation Analysis. We hypothesize that a hybrid-delivery ECD intervention will be lower cost, but remote interactions among participants may be an inferior substitute for in-person visits, leaving open the question of the most cost-effective program. Discussion Our goal is to determine the best model to maximize the intervention’s reach and sustained impacts to improve child outcomes. By integrating delivery into the ongoing operations of local Community Health Promoters (CHPs) within Kenya’s rural health care system, and utilizing new low-cost technology, our project has the potential to make important contributions towards discovering potentially scalable, sustainable solutions for resource-limited settings. Trial Registration NCT06140017 (02/08/2024) AEARCTR0012704.

## Trial Registration

NCT06140017 (02/08/2024) AEARCTR0012704.

## 1. Background

### 1.1 Background and rationale

An estimated 250 million children (43%) under age 5 in low- and middle-income country (LMIC) settings experience compromised cognitive and socioemotional development due to poverty, poor nutrition, and inadequate psychosocial stimulation (1). Myriad parenting interventions that promote responsive stimulation and early learning have been shown to be effective at improving early childhood development (ECD) outcomes in many LMIC settings (2, 3) at least in the short-term, but they are a) still too expensive to implement at scale in low-resource settings, especially in rural areas that lack resources and infrastructure to implement public health programs such as rural Kenya, and b) their early impacts tend to fade over time in the absence of continued support (4). New ways to deliver effective ECD parenting interventions in low-resource settings are sorely needed that are low-cost to be potentially scalable, while also able to sustain impacts over time.

The increasingly widespread use and low costs of mobile phones have spurred development of myriad mobile health (mHealth) interventions as potentially scalable means to deliver healthcare services (5) and improve health outcomes (6–8) in LMICs. There is growing evidence that mHealth interventions can also increase parental engagement (9–14), though all these studies come from high-income countries and focus primarily on parental literacy activities through simple SMS over short program durations of up to six months. To our knowledge, only one of the previous studies, York, Loeb, and Doss (2019b), found positive effects on children's early literacy.

While SMS-based interventions are cost-effective, they often fail to effectively communicate complex behavioral change messages, particularly in areas with lower literacy or in rural settings. This limitation is underscored by a recent study from Latin America that highlights the challenges in transitioning ECD programs to remote delivery during the COVID-19 pandemic (15). Moreover, while earlier SMS studies predominantly utilized passive, one-way text messages, recent recommendations advocate for the use of two-way and group interactions to provide more continuous support in mHealth behavior change programs (8, 16). The emerging trials in Peru and Brazil that incorporate digital tools to deliver content via two-way communications are indicative of growing efforts in this direction (17, 18). Despite these advancements, no prior ECD study has: 1) integrated audio-video content that extends beyond simple SMS, along with two-way and group interactions on digital platforms; 2) assessed the sustainability or cost-effectiveness of mHealth compared to in-person delivery; 3) examined impacts on a broader spectrum of children's developmental outcomes beyond literacy; and/or 4) been tested in an LMIC setting in an effectiveness trial with a large sample.

In a previous study, we provided scientific evidence that an 8-month ECD parenting intervention featuring fortnightly in-person group meetings delivered by Community Health Promoters (CHPs) from Kenya's rural health care system significantly improved child cognitive, language, and socioemotional development as well as parenting practices (19). The intervention's group-based model was also the most cost-effective among the few previous ECD interventions reporting costs (20). These results support the notion that health services -- particularly via the work of CHPs -- are ideal starting points to scale effective ECD interventions (21, 22). In a two-year follow-up assessment, we found that impacts were still positive, but smaller in magnitude (23), suggesting that a cost-effective program may still be too expensive for scaling in a rural LMIC setting such as rural Kenya, where health services are often underfunded.

Our proposed study addresses two key remaining questions in the ECD literature: 1) how to scale promising ECD programs in low-resource settings? and 2) how to sustain early impacts longer-term in a cost-effective way? We will test whether an mHealth-based intervention, that increasingly substitutes in-person meetings for remote delivery over time, can simultaneously achieve the competing goals of scalability and sustainability of ECD parenting interventions in LMICs.

## 1.2 Objectives and Research Questions

Our study aims to experimentally test the relative effectiveness and costs of a traditional in-person delivery model against a hybrid delivery model combining in-person meetings with remote mHealth delivery. This evidence-based ECD parenting intervention targets mothers and their children aged 6–18 months in rural Western Kenya, and originally lasted 8 months. Our primary goal is to determine the best model to maximize the intervention's reach and sustained impacts to improve child outcomes. By extending the original intervention over two years, we aim to enhance the program's ability to sustain improvements in parenting behaviors and children's outcomes over the long term. By integrating delivery into the ongoing operations of local CHPs within Kenya's rural health care system, utilizing mobile technology, and engaging national and local ECD policymakers and stakeholders as key collaborators from the project's inception, our project aims to contribute significantly towards discovering scalable, sustainable solutions for resource-limited settings. Our primary outcomes will include children's development, parental responsive stimulation, positive parenting behaviors, as well as maternal and family wellbeing.

Our research questions are:

1. Will an ECD responsive parenting curriculum adapted to mHealth delivery and tailored to the local cultural context be accepted by program beneficiaries and delivery agents?
2. What is the effectiveness and relative effectiveness in the short-term of a hybrid delivery model that increasingly substitutes remote mHealth delivery for in-person meetings compared to a traditional in-person delivery model?
3. Can a hybrid delivery model sustain early impacts in the medium-term better than a traditional in-person delivery model?
4. Are the cost-savings entailed in hybrid delivery large enough to make it more cost-effective than an in-person delivery model in the short- and medium-term?
5. What are the key implementation processes that can make a hybrid delivery more scalable than a traditional in-person delivery model?

To answer the research questions, we will collect measures of parental behaviors, knowledge, beliefs, self-efficacy, and mental health, together with child developmental outcomes at baseline, 12 and 24 months after the start of the interventions to assess short-and medium-term impacts. We will also track all program costs, by treatment arm, as well as private opportunity costs for delivery agents and participants, to estimate the relative short- and medium-term cost-effectiveness of the two delivery models under a societal perspective. A planned process evaluation will collect output measures of delivery and training quality, as well as attendance to in-person meetings and engagement in remote delivery, to uncover what aspects of the hybrid/remote delivery worked best and to inform a potential transition to scale.

### 2. Methods

## 2.1. Study Setting

This study will take place in rural areas of Kisumu and Vihiga counties in western Kenya, characterized by high rates of poverty, child mortality, and stunting (31–34%). We will select a total of three subcounties, all of which are large enough to select a total of 30 rural villages to participate into this study: Vihiga and Hamisi subcounties from Vihiga county, and Kisumu West subcounty from Kisumu county. All areas outside Kisumu town are predominantly rural, and our local NGO implementing partner, the Safe Water and AIDS Project (SWAP), has a local Jamii ("community") center in Vihiga county that will facilitate local monitoring and supervisory capacity. Most villagers are subsistence farmers or informal manual laborers. Despite their poverty, in a Fall 2021 survey in these areas 94% of households reported access to a mobile phone or smartphone, reflecting the vast expansion in mobile phone ownership worldwide. However, most phones are commonly owned by the household and often under the direct control of the husband or male household head.

## 2.2. Eligibility Criteria

This research project will involve a total of 1260 Kenyan mothers or other primary caretakers (1200 randomly selected for the main trial and 60 for the pilot study) and their children aged 6–18 months from 96 total villages located across rural areas of Kisumu and Vihiga counties in western Kenya. Within selected villages, eligible mother-child dyads will be defined by 1) mothers or other primary caretakers aged 18 years or older, and 2) with a child aged 6–18 months at recruitment without signs of severe mental or physical impairments. If the mother has more than one child aged 6–18 months at baseline, we will invite the youngest to participate. If the primary caretaker of an otherwise eligible child is the father or another male relative, he will be eligible for inclusion in our study, though we expect the vast majority of primary caretakers of children in this age range will be women, predominantly mothers. For simplicity we refer to this group as mothers.

## 2.3. Overview of the Trial Design

Our evaluation design is a cluster Randomized Controlled Trial (cRCT) stratified across three subcounties in rural western Kenya, implemented across 90 villages and 1,200 households. In this design, 90 CHPs and their associated villages will be randomly assigned to one of three treatment arms: 1) the in-person delivery model (Arm 1), where 30 CHPs will deliver a traditional in-person group-based intervention featuring a first intensive phase of 20 fortnightly village sessions over 12 months, followed by a less intensive second phase of monthly booster meetings for 12 additional months; 2) the mHealth delivery model (Arm 2), where 30 CHPs will deliver a hybrid intervention that increasingly substitutes in-person meetings with remote delivery over time; and 3) a control group (Arm 3), where 30 villages will continue to receive CHP services as usual. Interventions in Arms 1 and 2 will deliver the same content, based on a curriculum tested in an earlier trial (19, 24), but extended over two years to maximize its potential to sustain impacts.

In collaboration with the local NGOs the Safe Water and Aids Project (SWAP) and the ECD Network for Kenya (ECDNeK), we will train these 90 CHPs to implement the interventions in their respective villages. We will use a Training of the Trainers (TOT) model, where our core study team first trains SWAP's and ECDNeK's staff on the program to become lead trainers, and then these trainers will train cohorts of CHPs (defined by subcounty and arm) in the local language for the upcoming sessions. This local capacity-building TOT model will allow CHPs assigned to deliver in-person sessions to receive continuous training support from SWAP's and ECDNeK's staff through monthly local refresher trainings in each subcounty to prepare for the upcoming meetings.

The initial training for the early subcounty cohort will last 5 days and cover the first 4 sessions (whether delivered remotely or in-person), with approximately 20 CHPs assigned to Arms 1 or 2 in a given subcounty attending. After the first training, CHPs will host the first 4 sessions in their villages. Subsequent trainings for later sessions will be split by study arms within each subcounty due to the different delivery methods involved. We anticipate a total of five 1-week training sessions every two months, plus five monthly refreshers to cover Phase 1 of the intervention comprising 20 fortnightly sessions over 12 months. For the Phase 2 intervention comprising 12 monthly boosters, we anticipate three one-week training sessions, each covering boosters 1 to 4, 5 to 8, and 9 to 12. Figure 1 summarizes our study's evaluation design, the envisioned activities, and timeline.

### 2.4. Village and participant enrolment randomization strategies

We will randomly assign villages and households to the interventions in three steps. First, we will work with local administrative data to list all the potential study villages within each of the subcounties of Kisumu West (Kisumu County) and Vihiga and Hamisi (Vihiga County), estimated to have at least 8 households with children aged 6–18 months. SWAP will record the GPS coordinates of each village center (usually a church or marketplace). We will randomly sample 90 villages from this list, stratified by subcounty and with a minimum distance from all other sampled villages to minimize potential crossvillage contamination. Villages will comprise our study's clusters, from which we will sample households to participate in the study

Second, within each sampled village, we will conduct a census to create a full listing of all eligible households and record their GPS coordinates to facilitate collection of surveys. SWAP will train CHPs from the 90 selected villages to collect this basic information. Our previous experience collecting census data from villages in these subcounties show that most villages are rather small, with a size ranging from 8 to 16 households with a child 6–18 months of age. Therefore, we will invite all eligible households from selected villages to be part of the study. Our previous experience also shows minimal rates of refusal. Eligible participants will meet the criteria outlined above. Using the list of study participants per village and the recorded GPS coordinates, the village CHP will guide a trained interviewer to visit the households to invite eligible mother-child dyads into the study and undergo informed consent procedures for participation.

Third, after the baseline survey is completed, we will randomly assign CHPs and their associated villages to one of the two treatment arms or the control group. Each study arm is expected to have 30 CHPs and 400 households. CHPs in villages assigned to an intervention arm will attend the training course described above. Households assigned to an intervention arm will be contacted and invited to attend the ECD village sessions. All randomizations will be stratified by subcounty to ensure balance across treatment arms on any village-level characteristics that have the potential to have some relationship with intervention effects. We will pay all CHVs a stipend for their collaboration in the census and the intervention as appropriate.

## 2.5. Interventions

The ECD parenting interventions (Msingi Bora mHealth interventions hereafter) will build on our team's previous work in the Msingi Bora trial (24), including a subsequent booster extension featuring bi-monthly booster sessions (23). Msingi Bora's structured curriculum of 16 biweekly sessions was organized around five key messages: love and respect within the family, responsive talk, responsive play, hygiene, and nutrition, which were summarized to participants as love, talk, play, wash, food. Every fourth session served as review to help consolidate learning. Two updated curriculum manuals, one for in-person and one for hybrid, were available in the local languages for CHPs to use during training and delivery. Boosters had the same structure as earlier sessions, but focused on reinforcing language development and positive parenting strategies to manage children's behaviors.

For the Msingi Bora mHealth trial, the basic structure remains the same but additional materials will be incorporated to enhance areas like parental nutrition education and maternal wellbeing. The interventions will span two years and comprise two phases: Phase 1 will include 20 fortnightly sessions delivered over 12 months in villages assigned to a treatment arm, while Phase 2 will feature 12 monthly booster meetings. The target population is families with children aged 6–18 months at baseline, with mothers and their age-eligible child invited to participate in all sessions.

The intervention delivery will vary across treatment arms as follows:

#### Arm 1: In-person group sessions

In villages assigned to Arm 1, the first phase will feature 20 in-person group sessions delivered biweekly over 12 months by CHPs in their villages. Each session will last 60–90 minutes and cover one of five key messages: love and respect in the family, responsive play, responsive communication, hygiene, and nutrition. The inaugural session introduces the ECD program. Four sessions emphasize fostering love and respect within the family and maternal wellbeing, incorporating group discussions and role-playing to bolster maternal self-efficacy, self-esteem, and healthy family dynamics. Seven sessions will be devoted to responsive interactions in play and communication, where caregivers are shown how to play with children using games and materials available at home (such as a cup, bowl, and stones), and how to converse, sing and tell stories with the child to encourage language development. One session specifically addresses child health care practices, including diet and hygiene, though these topics are integrated into other sessions as well. Every group session, regardless of the session topic, will include 30 minutes of guided mother-child play and communication activities to reinforce new behaviors. Every fourth session serves as a review to consolidate learning. (five review sessions in total).

Phase 2 extends the program with 12 monthly booster sessions designed to help sustain improvements in parenting behaviors and children's outcomes over time. Boosters will hae the same structure and duration of Phase 1's sessions, but will focus on advanced strategies for responsive play and talk as children grow more capable, positive disciplinary practices to manage children's behaviors, as well as maternal mental health. Every third booster session will serve as a group review session that in addition will revisit hygiene and nutrition practices. New content will be introduced through group discussions, skits, and guided mother-child interactions.

#### Arm 2: mHealth hybrid delivery model

In villages assigned to Arm 2, the curriculum mirrors that of Arm 1, but integrates a hybrid mHealth delivery model, where most group sessions will be adapted to remote delivery via smartphones. Mothers in this arm will be provided with smartphones and a small monthly data plan to enable video content and engage in WhatsApp group interactions with other mother participants and CHPs, bolstering social networks of support and opportunities for social learning. Remote sessions will feature video demonstrations of play and communication activities by lead trainers, accompanied by audio recordings summarizing key points and offering guidance for how to enact these activities at home. A remote package containing these videos and audios will be distributed at the start of each session period, allowing mothers ample time to engage with the material.

The creation of village WhatsApp groups including the CHP is intended to facilitate follow-up on the enactment of new behaviors at home and to encourage mothers to share their experiences with other mothers, fostering social support networks. The Q&A activity at the end of each in-person session in Arm 1 will be replicated through WhatsApp group calls hosted by the CHP near the end of the session period.

The goal of this activity is to ensure barriers to behavior adoption are addressed, as well as discuss homework for the next session. Review sessions in Arm 2 will remain in-person to maintain some face-to-face interaction and adherence to the program.

### 2.6. Outcomes

The survey measures chosen for the assessment battery and how they relate to primary and secondary outcomes of interest are shown in Table 1. Most of these measures have already been validated, translated into Swahili and Luo using standard translation and back-translation methods, and used to evaluate short- and medium-term impacts in our earlier trial in the same study setting (19, 25). Most measures, except for those that are only applicable to children older than 2 years old, will be included in the assessment battery administered at each time point, including the Bayley III scale to assess children up to 42 months, and the Global Scales for Early Development short-form (GSED).

Table 1
Primary and Secondary Outcomes of Interest and Survey Measures

| Outcome of interest | Measure(s) | BL | ML | EL |
|---|---|---|---|---|
| Primary: Child cognition, language, executive function, socio-emotional | The Bayley Scales of Infant Development 3rd edition (Bayley's III) (26), validated in African settings and provides measures for all these dimensions of child development up to 42 months of age. At baseline we will only use the Bayley Cognitive scale. At midline and endline surveys we will add the Bayley Receptive and Expressive language scales. | X | X | X |
| | The Global Scales for Early Development v1.0 (GSED), short form, is a global assessment of child development valid for children up to 36 months old, recently developed by the WHO. It relies on caregiver report and exhibits good psychometric properties (27). | X | X | X |
| | The Caregiver Reported Early Development Instruments (CREDI) long form is a culturally and linguistically neutral set of questions that result in a summary of the overall developmental status of children up to 36 months (28). | | X | X |
| | The Wolke Scale (29) is an observational scale of children's behavior measuring approach, emotional tone, cooperation, vocalization, emotional security, and exploration previously used by our team. It's valid for children up to 5 years old. | | X | X |
| | The Strengths and Difficulties Questionnaire provides additional measures of emotional symptoms, conduct problems, and prosocial behavior among others (30, 31). This questionnaire is valid for children and young people 2–17 years old. | | X | X |
| | The WPPSI-III Wechsler Preschool & Primary Scale of Intelligence 3rd edition measures two verbal and two non-verbal cognitive abilities for children between the ages of 2 years, 6 months and 7 years, 7 months (32). | | X | X |
| | We will measuring executive functions administering four subtests of the International Development and Early Learning Assessment (IDELA): the Forward digit span (FDST), the Backward digit span (BDST), the Head, toes, knees and shoulder task (HTKS), and the Pencil tapping task (PTT) (33). Valid for children 3.5 to 6 years. | | X | X |
| Primary: Maternal stimulation practices | The Home Observation for Measurement of the Environment (HOME) inventory is a gold-standard measure of the quality and quantity of stimulation provided a child in the home: learning/play activities, availability of play materials, and parental warmth and disciplinary practices (34). Both at baseline and midline, we will use the 45-item version for children under age 3 years that combines 30 items on observed behaviors by the interviewer and 15 items based on maternal report. At endline, we will use a modification of the original tool for children 3–5 years old | X | X | X |

| Outcome of interest | Measure(s) | BL | ML | EL |
|---|---|---|---|---|
| | that includes 55 items, 30 of which are observed and the other 25 are reported by the mother. | | | |
| | The Family Care Indicators (FCI) (35) is an inventory of yes-no questions that measure parental investments in the following dimensions: Varieties of play materials (7 items), and play activities (6 items). | | X | X |
| Primary: Maternal mental health | Depressive symptoms will be assessed the Patient Health Questionnaire (PHQ-8), a scale widely used and validated in African countries (36). Parental stress will be assessed using the Parenting Stress Index (PSI) (37). | X | X | X |
| Primary: Social networks of support | Networks of support are likely to change in size, composition, and quality, as a consequence of the intervention, and these changes can mediate parenting behavioral changes. Using standard methods to identify networks in rural areas (38, 39), we'll ask mothers to name up to 10 villagers, including study participants, they feel connected with to discuss their children's health and family issues. | X | X | X |
| Secondary: Child anthropometrics | Child weight and height, stunting, and arm circumference will be measured using techniques for the WHO Multicenter Growth Reference Study (MGRS) (40). At Baseline we will only collect height. At Midline and Endline all the measures will be collected. | X | X | X |
| Secondary: Nutrition practices | Dietary diversity will be measured by maternal self-report of the foods eaten by the child in the last 24 hours, following WHO recommendations about young and infant child feeding (41). | X | X | X |
| Secondary: Preventive health | A composite score of nine items including access to safe water, use of latrines, immunizations against illnesses like diphtheria, polio, tetanus, and others, deworming, etc. (42). | X | X | X |
| Secondary: Household decision-making and IPV | Questions about whether only the mother, only the husband/partner or both jointly as a couple make decisions about key household decisions, as well as maternal beliefs and experience of Intimate Partner Violence (IPV), will be borrowed from the widely used Demographic Health Surveys (DHS), which are collected for Kenya every few years (last one being from 2022 (43). | X | X | X |
| Secondary: Knowledge | Mother’s knowledge of child development will involve six questions asking mothers at what age children generally acquire social and cognitive skills (42). | X | X | X |
| Secondary: Self-efficacy | A set of two-part questions where first we ask the mother if she is able to provide certain playthings or new games to stimulate their children, and if they respond yes, how confident they feel they will continue doing it in the future. The set of 15 questions are based on items of the HOME inventory, which is the gold standard measure of responsive parenting practices. | X | X | X |

| Outcome of interest | Measure(s) | BL | ML | EL |
|---|---|---|---|---|
| Secondary: Maternal Problem Solving and Learning by doing | We will administer the Tower of London test (44), a test that consists of two boards with pegs and several beads with different colors developed to measure executive functions related to planning abilities. By increasing the complexity of this task, we can also assess persistence, which is an important factor of learning by doing. Problem solving and learning by doing are two potential pathways through which the interventions can persistently improve parenting behaviors (45). | X | X | X |

## 2.7. Blinding

Our study will have separate teams for collection of surveys and program implementation. Interventions will be coordinated by the implementation team at SWAP led by Co-I Alu, which will be in charge of leading the TOT training of CHPs and monitoring the quality of implementation activities. This team will also coordinate the work of 3 subcounty teams, each composed by a subcounty supervisor and 2 mentor CHPs, which will collect attendance and monitoring data, and supervise the work of the CHPs on a daily basis. Survey data collection will be conducted by an external team of qualified enumerators and supervisors hired and supervised by a second evaluation team at SWAP led by Mr. Odhiambo, which will only be involved in evaluation activities. Our core team of investigators will directly train enumerators into the household survey and the child assessments. Due to the nature of the intervention, the participants and delivery agents will not be blinded to their study allocation as part of the program implementation team. Survey enumerators will, however, be blinded to the intervention allocation status of participants and villages. Baseline surveys will be collected prior to randomization.

## 2.8. Compliance

We do not anticipate noncompliance with treatment status for those villages and households assigned to a control arm because our sampling frame will ensure a healthy minimum distance between villages and CHPs included in the study. For individual households in villages assigned to a treatment arm, our power calculations presented below account for noncompliance with treatment by including an expected attendance rate of 75% to the sessions.

## 2.9. Retention

Once a mother-child dyad is enrolled into the study, we will make every reasonable effort to follow the dyad for the entire study period. Both the baseline and midline surveys will collect mobile phone numbers for household members to facilitate the tracking in subsequent surveys as well as invitations to attend sessions, if appropriate. The mobile number of one neighbor will additionally be collected to help identify cases of non-retention. Reasons for non-retention include migration to another village or subcounty due to separation, (re)marriage, and relocation for work. We will record these cases, including the new address and contact information, and will follow-up these families at midline and endline. At each survey round we will make up to 4 attempts to contact a household for resurveying prior to dropping from the sample. Our power calculations account for 7% annual attrition to allow for such instances.

## 3. Data

### 3.1. Sample size and power calculations

This cluster RCT will involve a total of 1200 Kenyan mothers-children dyads which result in enough power to identify impacts on our primary outcomes. Our power is calculated for our primary outcome of children's cognitive development using the Bayley III scale, which has a usual mean of 100 with a standard deviation (SD) of 15. The Msingi Bora trial had an effect size on children's cognitive scores of 0.52 SD, and an annual attrition rate of 7%. We had an average of 75% compliance among mothers throughout biweekly sessions and boosters, with an intra-cluster correlation coefficient (ICC) of 0.02 from Vihiga county. Assuming 80% power, 75% compliance, an annual attrition rate of 7%, a more conservative ICC of 0.04, and after correcting by baseline covariates, with 30 villages (400 mother-child dyads) per treatment arm, at midline we would be able to detect a difference of at least 0.22 SD in cognition between the in-person versus mHealth intervention arms, or between any intervention arm and the control group. We will use the step-down method of Romano and Wolf to adjust the p-values for multiple hypothesis testing (46).[8] Under these assumptions, the minimum detectable effect would lie between 0.25–0.27 SD. At endline, assuming a 15% of accumulated attrition, in side-by-side comparison between treatment arms we would be able to detect a difference in child cognition of 0.25 SD. Adjusting for multiple hypothesis testing, the minimum detectable effect would lie between 0.29–0.31 SD. Finally, to improve the robustness of our estimated impacts for individual outcomes, we will construct indices of child and parental outcomes estimated with latent factor models and estimate the intervention effects on these indices. We anticipate at least four indices representing different families of outcomes: i) child developmental measures; ii) parental stimulation and health behaviors; iii) parental knowledge and beliefs; and iv) parental wellbeing.

### 3.2. Data collection

#### Household surveys and procedures

For the 1,200 respondent mothers and children recruited for the main trial, participation will involve a 60–90-minute baseline survey. Two trained interviewers will visit households to invite mothers into the study and to undergo informed consent procedures for participation. All households, irrespective of their village's eventual group assignment, will be asked to provide written or verbal consent explaining the purpose and contents of the study as well as their anticipated time commitment for attending the village-based sessions and/or participating in sessions delivered remotely, if their villages are assigned to an intervention arm. Mothers will be made clear that participation in the surveys is voluntary and participation in the intervention is not guaranteed but based on their village's random assignment. For those households that express a willingness to continue in the study, in a first visit one interviewer will conduct the maternal and socioeconomic surveys, and in a second visit another interviewer will assess the child.

All households surveyed at baseline will be re-contacted to undergo a midline survey roughly 15 months later, immediately after the end of Phase 1's intervention, to assess short-term impacts after 12 months. Duration, procedures and measures will be identical to baseline. The interviewer will reassess the child assessed at baseline and re-interview the mother. We will also conduct an endline survey at the end of the two-year interventions to evaluate medium-term impacts. The midline and endline surveys will assess the same maternal and children's outcomes as at baseline. However, in the last two surveys we will include new measures of children's cognitive, socioemotional, and executive functioning development that are only applicable to children older than 2 years old (see Table 1). As at baseline, a team of two enumerators will conduct the fieldwork, one for the socioeconomic and maternal surveys, and the other to assess the child. Enumerators will be masked to intervention assignment. All study households will receive a thank you gift consisting of a hygiene pack valued at 400 Ksh for completion of each survey wave.

#### Monitoring and process data

We will collect both qualitative and quantitative data on the quality and fidelity of delivery following the CARE (Consolidated Advice for Reporting ECD implementation research) guidelines (47). A planned collection of monitoring data will account for the implementation differences by arm. For the in-person sessions, subcounty supervisors will collect detailed implementation data in the form of attendance sheets, monitoring checklists measuring CHP's quality of delivery, as well as CHP's self-assessment forms. For the remote sessions, we will continue to collect monitoring checklists and CHP self-assessment forms, but focused on the CHPs' performance during WhatsApp group calls. In addition, subcounty supervisors will collect a parental remote engagement form completed by CHPs in Arm 2 at the end of each remote session, measuring individual-level measures of parental engagement with the audiovisual content, and participation in the WhatsApp group calls and chats. All the data will be collected using SurveyCTO and will be transmitted to SWAP servers in Kisumu, where SWAP staff will clean and aggregate the data to be transferred to an aggregate server hosted at USC. Finally, following the end of all interventions, local research staff will be trained to conduct FGDs with a minimum of 20 mothers and 12 CHPs assigned to the 2 intervention arms. The exit FGDs will aim to learn what worked and what did not from CHPs' and parents' perspectives, and this qualitative data can be used to explain quantitative findings using mixed methods.

#### Costing Data

As explained above, our cost-effectiveness analysis is a key project aim. For a comprehensive understanding of the project's costs, we will adopt a societal perspective for this analysis that includes both provider's implementation costs (e.g., CHP payments, cost of SMS) and opportunity costs to the household and community (e.g., time costs of delivering and attending sessions, and interacting remotely, for CHPs and mothers, respectively), separately by intervention arm. We will track all implementation costs during Phases 1 and 2, by treatment arm, using a step-down accounting cost method based on actual incurred costs provided by SWAP's financial statements. We will use economic costing methods to estimate opportunity costs for mothers and CHPs as appropriate. We will include additional opportunity costs stemming from maternal behavior changes induced by the interventions. We will collect and report all costs in accordance with the Consolidated Health Economic Evaluation Reporting Standards (CHEERS) guidelines (48).

#### Data Management

All data collected will include personable identifiable information (PII), but it will be coded so only a household identifier can be linked to PII. Surveys will be collected via tablets and contain personal identifiers (names), anthropometric and psychosocial measures of children and their mothers, and mobile telephone numbers. Data from Tablet-based surveys will be safely stored in SurveyCTO and will be downloaded and analyzed using Stata software version 16 (College Station, TX). To increase security over paper questionnaires, these will be encrypted in SurveyCTO. Participant names will be removed from the data and no longer stored in any tablet after the successful linking of the midline and endline surveys to baseline data using a USC-generated ID. Access to this linked file will be restricted only to authorized study staff. Data transfer from SWAP to USC will be done with only encrypted, password-protected files. Survey data will be treated with the maximum norms of confidentiality following the study protocols involving human subjects reviewed by the USC's Institutional Review Board IRB) as well as the Maseno University Ethical Review Committee(MUERC).

### 3.3. Statistical Methods

#### Short-term Impacts

We will use outcomes data from the midline survey and our cluster randomized design to estimate the short-term effectiveness of the two intervention arms relative to the control Arm 3. We estimate relative effectiveness in an Intention-to-Treat (ITT) framework. Let $Y$ denote an outcome of interest at midline, and D is a vector of dummy variables for the random allocation to one of the treatment arms: In-person model $(D_1)$, Hybrid model $(D_2)$, and the Control group $(C)$. The ITT parameters capturing the intervention effects relative to the control group can be estimated from the following linear regression:

$$Y = \alpha_0 + \alpha_1^{ITT} D_1 + \alpha_2^{ITT} D_2 + X' \beta + \lambda + \epsilon \qquad [1]$$

In equation [1], $\alpha_k^{ITT}$ is the parameter capturing the ITT impact of intervention type $\{k = 1,2\}$ relative to the control group on the final outcome; $\mathrm{X}$ is a vector of covariates that includes children's age and sex, family socioeconomic status; and outcomes at baseline; $\lambda$ is the randomization strata (the subcounty); and $\epsilon$ is an error term, clustered at the village level. The ITT parameters are identified by the orthogonality between the error term and treatment status. We will correct for multiple hypothesis testing among potentially highly correlated outcomes using the Romano-Wolf estimator (46).

Similarly, we can estimate the Treatment-on-the-Treated (TOT) parameter that captures the average treatment on participants of treatment arm $k$ with respect to the control group using the following Two-Stage Least Squares procedure:

$$Y = \alpha_0 + \alpha_1^{TOT} P_1 + \alpha_2^{TOT} P_2 + X' \beta + \epsilon \qquad [2]$$

$$P_1 = b_0 + b_1 D_1 + b_2 D_2 + X' \gamma + \eta \quad [3]$$

$$P_2 = c_0 + c_1 D_1 + c_2 D_2 + X' \delta + \pi \quad [4]$$

In equation [2], $\alpha_k^{TOT}$ is the TOT impact of intervention $\mathrm{k}$ on the outcome, and $\{P_1,\ P_2\}$ are dummy variables for observed participation in the in-person and hybrid interventions, respectively, which can differ from the random allocation if there is imperfect compliance. Equations [3] and [4] correct for selection bias into participation by modelling the participation decision using the randomization as an instrumental variable and estimating by 2SLS methods.

#### Medium-term Impacts

Assessing medium-term impacts of the interventions at endline is straightforward and draws from the same analysis plan outlined through equations [1]-[4] for short-term impacts. Instead of using the outcomes from the midline survey, we use outcomes measured at the final endline survey.

#### Cost-effectiveness

Following recent guidelines for cost-effectiveness analyses, we will calculate incremental cost-effectiveness ratios (ICER) expressed in terms of incremental ITT impacts in child outcomes per $100 investment. For example, we can calculate the ICER for the hybrid intervention relative to the in-person intervention with the following formula:

$$\mathrm{CER} = \frac{(\alpha_2^{\mathrm{ITT}} - \alpha_1^{\mathrm{ITT}})*100}{\mu_{\mathrm{c}2} - \mu_{\mathrm{c}1}} \quad [5]$$

where $\mu_{\mathrm{c}2}$ is the cost per child of the hybrid intervention in Arm 2, $\mu_{\mathrm{c}1}$ is the cost per child of the in-person intervention in Arm 1, $\alpha_2^{\mathrm{ITT}}$ is the ITT impact of Arm 2 in an outcome of interest, and $\alpha_1^{\mathrm{ITT}}$ is the analogous ITT impact in Arm 1.

#### Mediation Analysis

To examine the interventions' pathways of change we will conduct a Mediation Analysis following a Monte Carlo simulations approach (49). In a standard mediation model where the outcome of interest is $Y$ and the mediating factor is $M$, the goal is to estimate the magnitude and significance of the intervention's indirect effect $(a * b)$ as opposed to the direct effect $(c)$ from the following model:

$$Y = b_0 + \mathrm{b}M + cD + \epsilon \qquad [6]$$

$$M = a_0 + aD + u \qquad [7]$$

Using this simple model, we can investigate the pathways through which one of our intervention arms influences changes in a specified outcome of interest. For example, we can explore if intervention impacts on children's outcomes $(Y)$ are going through changes in mediators of change $(M)$ such as stimulation behaviors, disciplinary practices, nutrition practices, or through other maternal intermediate outcomes including knowledge, self-efficacy, social networks, or mental health. To do this, we will perform the following steps. First, we will run regressions using equation [7] for each potential mediator of interest to estimate the intervention impact on the mediator, captured by the coefficient $\widehat{a}$. Second, for each potential mediator, we will run regressions using equation [6] including treatment dummies and on the particular mediator of interest, to estimate the coefficient $\hat{b}$. Using the estimated regression coefficients and their standard errors, we will compute the 95% Monte Carlo confidence intervals for the indirect effect $\left(\widehat{a} * \hat{b}\right)$ based on a very large number of repetitions. An interval that does not include zero indicates a significant indirect effect of that particular mediating variable. To assess the total indirect effect including all the relevant mediators, we look at the Monte Carlo confidence intervals using the paths a and b from all mediators that resulted to be significant individually but now included together in the same regression model, as in equation [6].

##### Heterogeneous Effects

Given the complexity of our experiment and the number of hypothesized channels through which our interventions may affect final outcomes, it is challenging to ex ante hypothesize all possible heterogeneous effects. However, to inform the design of targeted policies and address equity-efficiency considerations by remediating socio-economic gaps in child development, it is important to understand whether our interventions are more effective among more disadvantaged households. Therefore, we plan to test for heterogeneous treatment effects by children's sex and age, maternal age an education, household wealth, and child outcomes at baseline.

##### Missing data and attrition

In all our analyses, we will handle missing data and attrition across survey waves by fitting logistic regression models to assess whether a missing observation is random. To correct for potential non-random attrition in all our regressions and in the calculation of standard errors, we will use of Inverse Probability Weighting (IPW) methods (50), reweighting our data in such a way that a larger weight is given to participants who are underrepresented in the midline or endline sample as a result of attrition. We will complement this strategy with the estimation of Lee Bounds for all our results (51). In order to test for the importance of outliers, we will check for the robustness of our estimates with the full sample by comparing the estimates from this sample with those from a sample cutting the bottom 2% and the top 2% and testing for the significance of this difference.

### 3.4. Adverse Effects

Interviews, surveys, and the ECD program are low-risk, and therefore adverse events (AEs) are very unlikely, and any experienced AEs will be likely due to factors unrelated to the study. However, there may be adverse consequences to participation that were unintended or unexpected (e.g., giving smartphones to women might trigger intra-household conflict). In these instances, we will rely on local monitoring and reporting by SWAP, which has vast experience handling fieldwork activities in community health projects. All SWAP's staff has been trained to report adverse events, as well as will intervene as necessary, assessing the participant's state, and developing an appropriate plan. Incident reports will be written within one business day and study investigators will inform the IRBs of all AEs. This plan has been reviewed and approved by the local IRB at Maseno University, as well as USC's IRB.

### 3.5. Dissemination of results

Our dissemination plan will consist of two central strategies:

1) Engagement with local ECD Policy: Our research team includes staff based out of both Kisumu and Nairobi, Kenya's capital, as well as planned activities in all years to ensure our project remains engaged and connected to the local ECD policy and interested stakeholders throughout its duration. In Year 1, Co-I Mwoma and her team at the ECD Network for Kenya (ECDNeK) will host full-day sensitization workshops in the city of Kisumu where 60 key County and National policy makers, stakeholders and other partner agencies will be invited to the launch of the project to ensure their input to the planning of project activities. Following this launch, we plan to create an advisory board with representatives from partner agencies and interested stakeholders (e.g., Ministry of Health, Ministry of Labor and Social Protection, Africa Early Childhood Network), who will meet (virtually) twice per year to get updates on project progress and provide feedback and guidance. ECDNeK will also employ a fulltime policy coordinator to coordinate project networking and advocacy activities and attend meetings to ensure our project's connection to the local ECD policy environment. Please see attached letters of support from various Ministry of Health (MOH) personnel.

2) Dissemination of Results: ECDNeK and SWAP will coordinate dissemination and policy engagement workshops in later project years to share project findings. Our research team will publish the study's protocol and all findings in peer-reviewed journals in economics and public health, as well as present findings at domestic and international conferences such as the Society of Research in Child Development (SRCD).

## 4. Discussion

There is an urgent need to discover the most effective and potentially scalable models of delivery for evidence-based responsive caregiving interventions that can improve children's developmental outcomes among disadvantaged children in resource-poor settings. There is also an urgent need to ensure those improvements are sustained in order to realize long-term benefits and help break the intergenerational transmission of poverty. There is now an abundance of evidence that ECD responsive caregiving programs can realize short-term impacts on parenting behaviors and children's outcomes.

The challenges now are to find ways to sustain these early impacts in the longer-term and to scale those programs.

Our adaptation and test of the Msingi Bora program for remote delivery via smartphones and our strategy to extend the interventions to complete two years of continued program support are meant to address both challenges head-on. Yet, our study might face a few practical and operational issues. The first one is related to the measurement of children's outcomes with reliable measures based on direct assessments and not on parental reports. For instance, internationally accepted "gold standard" direct assessments of child development, such as the Bayley-III and the WPPSI-IV, have been primarily developed for high-income country settings. These assessments are time-consuming, require highly skilled and extensively trained assessors, and necessitate the child to be in the right mood for the test, with the mother's presence often needed to comfort the child. To overcome these challenges, we will conduct a one-month training program for survey enumerators. This includes two weeks of in-house intensive training in Kisumu and practice with local children, one week of supervised practice with rural families, and a final week of field testing to establish test-retest and inter-rater reliability (IRR) measures before full implementation.

The second challenge relates to the risk of intervention spillover across villages in the cRCT study, especially for the remote intervention featuring video content. We will adopt several strategies to mitigate this risk. First, CHPs' catchment areas will be mapped and selected to ensure a minimum distance between sampled villages. Despite this, the risk cannot be entirely eliminated. Therefore, SWAP will work closely with Community Health Units to raise awareness among CHPs from both intervention arms and the control group about the importance of avoiding cross-village contamination and reporting any such instances. Second, CHPs will collect detailed attendance data, including the village of residence, at the beginning of every in-person session and WhatsApp group call. This will help identify and address any uninvited participants. Finally, during the sampling stage, SWAP will identify concurrent interventions from other NGOs in the pre-selected villages to avoid overlap whenever possible, and when overlap is unavoidable, document these interventions to incorporate this information into our statistical analyses.

Third, the remote nature of Arm 2 presents the challenge of technology illiteracy among our study participants, which may prevent families from downloading video content demonstrating the activities or joining WhatsApp group calls and chats. To mitigate this risk, we will conduct a special in-person session before the start of remote sessions in this arm to sensitize families and provide extensive training in the use of smartphones. This session will cover accessing audio and video content, using WhatsApp to interact with other mothers and CHPs in group chats, joining WhatsApp group calls, and downloading remote packages with audiovisual content from both WhatsApp and memory cards. These memory cards will contain all the audiovisual materials for all remote sessions. Additionally, we will provide internet packages to the families to facilitate access to the remote content and the virtual social network. Every three remote sessions, the fourth session will be in-person to maintain adherence to the program, encourage smartphone retention, and reinforce the main points of the shared content. Finally, we will implement a buddy system, where mothers living nearby each other will be encouraged to support each other in using smartphone technology, which can further improve program adherence and the enactment of new parenting behaviors.

In total, the strengths of our study outweigh the limitations.

## Abbreviations

ECD: Early Childhood Development cRCT: cluster Randomized Controlled Trial
CHP: Community Health Promoter mHealth: Mobile Health

LMIC: Low and Middle-Income Country
SWAP: Safe Water and AIDS Project

ECDNeK: ECD Network for Kenya

Ksh: Kenyan Shillings

NGO: Non-Governmental Organization

ITT: Intention to Treat

ATT: Average Treatment on the Treated
TOT: Training of the Trainers

SMS: Short Message Service
CER: Cost-Effectiveness Ratio

SD: Standard Deviation

FGD: Focus Group Discussion

## Declarations

#### Ethics approval and consent to participate

Study protocols have been reviewed and approved by the USC Institutional Review Board with study ID UP-22-00336, and the local Kenyan review board at the Maseno University Ethics Review Committee (MUERC) Approval MSU/DRPI/MUSERC/01201/23. We will obtain written and verbal consent from all study participants prior to enrollment. Important protocol modifications will be submitted to all IRBs for approval as amendments.

#### Consent for publication

Not applicable.

#### Availability of data and materials

The final dataset will include the child assessments merged to the self- reported demographic, behavioral and wellbeing survey data from mothers and the household. We will make this data publicly available by posting it in de-identified form to the Inter-university Consortium for Political and Social Research (ICPSR) data bank. This data bank is accepted by NIH and USC is a subscriber to it. More information on their data confidentiality practices can be found here: https://www.icpsr.umich.edu/web/pages/datamanagement/confidentiality/index.html.

#### Competing interests

The authors declare that they have no competing interests.

#### Funding

This work is supported by the Eunice Kennedy Shriver National Institute of Child Health & Human Development (NICHD) with award number R01HD107116. NICHD will play no role in the design, collection, analysis, or interpretation of the data nor decisions to submit for publication any resulting manuscripts.

#### Authors' contributions

ILG and JL conceptualized the study and drafted the manuscript protocol. FA and PJ reviewed and provided critical input to study design and conceptualization as well as the manuscript. All authors read and approved the final manuscript.

#### Acknowledgements

We thank Alie Eleveld, Alex Mwaki, Edith Alu, Aloyce Odhiambo, Ronald Otieno, Pamela Mota, Joab Ochieng, and the entire team at the Safe Water and AIDS Project (SWAP), as well as Dr. Teresa Mwoma, Sally Moraa, and the team at the ECD Network for Kenya, for their invaluable support in carrying out this study. We also extend our gratitude to Douglas Newball Ramirez for his invaluable assistance in implementing this study and writing this paper.

## References

1. Black MM, Walker SP, Fernald LC, others. Early childhood development coming of age: science through the life course. The Lancet. 2017;389:77–90.
2. Aboud FE, Yousafzai AK. Global health and development in early childhood. Annu Rev Psychol. 2015;66:433–57.
3. Jeong J, Franchett EE, de Oliveira C V, Rehmani K, Yousafzai AK. Parenting interventions to promote early child development in the first three years of life: A global systematic review and meta-analysis. PLoS Med. 2021;18(5):e1003602.
4. Jeong J, Pitchik HO, Fink G. Short-term, medium-term and long-term effects of early parenting interventions in low-and middle-income countries: a systematic review. BMJ Glob Health. 2021;6(3):e004067.
5. Kahn JG, Yang JS, Kahn JS. 'Mobile'health needs and opportunities in developing countries. Health Aff. 2010;29(2):252–8.
6. Hall CS, Fottrell E, Wilkinson S, Byass P. Assessing the impact of mHealth interventions in low-and middle-income countries--what has been shown to work? Glob Health Action. 2014;7(1):25606.
7. Aranda-Jan CB, Mohutsiwa-Dibe N, Loukanova S. Systematic review on what works, what does not work and why of implementation of mobile health (mHealth) projects in Africa. BMC Public Health. 2014;14(1):188.
8. Gurman TA, Rubin SE, Roess AA. Effectiveness of mHealth behavior change communication interventions in developing countries: a systematic review of the literature. J Health Commun. 2012;17(sup1):82–104.
9. Cortes KE, Fricke H, Loeb S, Song DS, York BN. Too little or too much? Actionable advice in an early-childhood text messaging experiment. MIT Press One Rogers Street, Cambridge, MA 02142-1209, USA journals-info~…; 2021.
10. Doss C, Fahle EM, Loeb S, York BN. More than just a nudge supporting kindergarten parents with differentiated and personalized text messages. Journal of Human Resources. 2019;54(3):567–603.
11. Mayer SE, Kalil A, Oreopoulos P, Gallegos S. Using Behavioral Insights to Increase Parental Engagement: The Parents and Children Together (PACT) Intervention. 2015;
12. Hurwitz LB, Lauricella AR, Hanson A, Raden A, Wartella E. Supporting Head Start parents: impact of a text message intervention on parent--child activity engagement. Early Child Dev Care. 2015;185(9):1373–89.
13. Bloomfield J, Balsa A, Cid A. Using behavioral insights in early childhood interventions: The effects of Crianza Positiva e-messaging program on parental investment. Rev Econ Househ. 2023;21(1):95–130.
14. York BN, Loeb S, Doss C. One step at a time: The effects of an early literacy text-messaging program for parents of preschoolers. Journal of Human Resources. 2019;54(3):537–66.
15. Rubio-Codina M, Lopez-Boo F. What Have We Learned from the Design and Delivery of Remote and Hybrid Early Childhood Development Services During the Pandemic? IDP Discussion Paper IDB-DP-00963. 2022 Aug.

16. Chiang N, Guo M, Amico KR, Atkins L, Lester RT, others. Interactive two-way mHealth interventions for improving medication adherence: an evaluation using the behaviour change wheel framework. JMIR Mhealth Uhealth. 2018;6(4):e9187.
17. Pena SMH, Mäusezahl D, Jäggi L, Aguilar L, Llatance MA, Castellanos A, et al. Digital Support Systems to Improve Child Health and Development in Peru: Protocol for a Randomized Controlled Trial. JMIR Res Protoc. 2023;12(1):e50371.
18. Solis-Cordero K, Marinho P, Camargo P, Takey S, Lerner R, Ponczek VP, et al. Effects of an online play-based parenting program on child development and the quality of caregiver-child interaction: a randomized controlled trial. In: Child & Youth Care Forum. 2023. p. 935–53.
19. Luoto JE, Lopez Garcia I, Aboud FE, Singla DR, Fernald LCH, Pitchik HO, et al. Group-based parenting interventions to promote child development in rural Kenya: a multi-arm, cluster-randomised community effectiveness trial. Lancet Glob Health. 2020;9(3):e309–19.
20. Lopez Garcia I, Saya U, Luoto JE. Cost-effectiveness and economic returns of group-based parenting interventions to promote early childhood development: Results from a randomized controlled trial in rural Kenya. PLoS Med. 2021 Sep 28;
21. Richter LM, Daelmans B, Lombardi J, Heymann J, Boo FL, Behrman JR, et al. Investing in the foundation of sustainable development: pathways to scale up for early childhood development. The Lancet. 2017;389(10064):103–18.
22. Tomlinson M, Hunt X, Rotheram-Borus M. Diffusing and scaling evidence-based interventions: eight lessons for early child development from the implementation of perinatal home visiting in South Africa. Ann N Y Acad Sci. 2018;1419(1):218–29.
23. Lopez Garcia I, Luoto J, Aboud F, Fernald L. Group Meetings and Boosters to Sustain Early Impacts on Child Development: Experimental Evidence from Kenya. 2023;
24. Luoto JE, Lopez Garcia I, Aboud FE, Fernald LCH, Singla DR. Testing means to scale early childhood development interventions in rural Kenya: the Msingi Bora cluster randomized controlled trial study design and protocol. BMC Public Health. 2019;19(1):259.
25. Lopez Garcia I, Fernald LCH, Aboud F, Luoto JE. Sustained Impacts of Msingi Bora, a group-based parenting Intervention to promote early childhood development in rural Kenya. Los Angeles; 2022 Feb.
26. Aylward GP. Bayley Scales of Infant and Toddler Development. Encyclopedia of Clinical Neuropsychology. 2011;357–8.
27. Cavallera V, Lancaster G, Gladstone M, Black MM, McCray G, Nizar A, et al. Protocol for validation of the Global Scales for Early Development (GSED) for children under 3 years of age in seven countries. BMJ Open. 2023;13(1):e062562.
28. McCoy DC, Sudfeld CR, Bellinger DC, Muhihi A, Ashery G, Weary TE, et al. Development and validation of an early childhood development scale for use in low-resourced settings. Popul Health Metr. 2017;15:1–18.

29. Wolke D, Skuse D, Mathisen B. Behavioral style in failure-to-thrive infants: a preliminary communication. J Pediatr Psychol. 1990;15(2):237–54.
30. Goodman R. Psychometric properties of the strengths and difficulties questionnaire. J Am Acad Child Adolesc Psychiatry. 2001;40(11):1337–45.
31. Goodman R. The Strengths and Difficulties Questionnaire: a research note. Journal of child psychology and psychiatry. 1997;38(5):581–6.
32. Wechsler D. Wppsi-iii. Wechsler Preschool and Primary Scale of Intelligence. 2004;
33. Pisani L, Borisova I, Dowd AJ. Developing and validating the international development and early learning assessment (IDELA). Int J Educ Res. 2018;91:1–15.
34. Caldwell BM, Bradley RH. Home inventory administration manual. University of Arkansas for Medical Sciences; 2003.
35. Hamadani JD, Tofail F, Hilaly A, Huda SN, Engle P, Grantham-McGregor SM. Use of family care indicators and their relationship with child development in Bangladesh. J Health Popul Nutr. 2010;28(1):23.
36. Löwe B, Kroenke K, Herzog W, Gräfe K. Measuring depression outcome with a brief self-report instrument: sensitivity to change of the Patient Health Questionnaire (PHQ-9). J Affect Disord. 2004;81(1):61–6.
37. Abidin RR. Parenting Stress Index:(Short Form). Pediatric Psychology Press Charlottesville, VA; 1990.
38. Maertens A, Barrett CB. Measuring social networks' effects on agricultural technology adoption. Am J Agric Econ. 2013;95(2):353–9.
39. den Broeck K, Dercon S. Information flows and social externalities in a Tanzanian banana growing village. J Dev Stud. 2011;47(2):231–52.
40. de Onis M, Garza C, Victora CG, Onyango AW, Frongillo EA, Martines J. The WHO Multicentre Growth Reference Study: planning, study design, and methodology. Food Nutr Bull. 2004;25(Supplement 1):15S----26S.
41. World Health Organization, UNICEF. Global strategy for infant and young child feeding. 2003.
42. Singla DR, Kumbakumba E, Aboud FE. Effects of a parenting intervention to address maternal psychological wellbeing and child development and growth in rural Uganda: a community-based, cluster-randomised trial. Lancet Glob Health. 2015;3(8):e458----e469.
43. Kenya National Bureau of Statistics. Kenya Demographic and Health Survey 2022, Key Indicators Report. Government of Kenya; 2023.
44. Phillips LH, Wynn VE, McPherson S, Gilhooly KJ. Mental planning and the Tower of London task. The Quarterly Journal of Experimental Psychology Section A. 2001;54(2):579–97.
45. Briscoe C, Aboud F. Behaviour change communication targeting four health behaviours in developing countries: a review of change techniques. Soc Sci Med. 2012;75(4):612–21.

46. Romano JP, Wolf M. Exact and approximate stepdown methods for multiple hypothesis testing. J Am Stat Assoc. 2005;100(469):94–108.
47. Yousafzai AK, Aboud F. Review of implementation processes for integrated nutrition and psychosocial stimulation interventions. Ann N Y Acad Sci. 2014;1308(1):33–45.
48. Husereau D, Drummond M, Petrou S, Carswell C, Moher D, Greenberg D, Augustovski F, Briggs AH, Mauskopf J LE. CHEERS: Consolidated Health Economic Evaluation Reporting\nStandards. Eur J Health Econ. 2013;14(3):367–72.
49. Preacher KJ, Selig JP. Advantages of Monte Carlo confidence intervals for indirect effects. Commun Methods Meas. 2012;6(2):77–98.
50. Robins JM, Rotnitzky A, Zhao LP. Estimation of regression coefficients when some regressors are not always observed. J Am Stat Assoc. 1994;89(427):846–66.
51. Lee DS. Training, wages, and sample selection: Estimating sharp bounds on treatment effects. Rev Econ Stud. 2009;76(3):1071–102.

## Footnotes

1. The Romano and Wolf is a stepwise multiple testing procedure that asymptotically controls the family-wise error rate. In this method, each coefficient is tested in a step-down fashion using bootstrapping methods allowing for the t-statistics of outcomes to be mutually dependent. Critical values are constructed based on the maximum t-statistic across all outcomes included in each iteration of the test. In the first iteration, all outcomes are included. In further iterations, only outcomes that did not pass the previous test are included.

## Figures

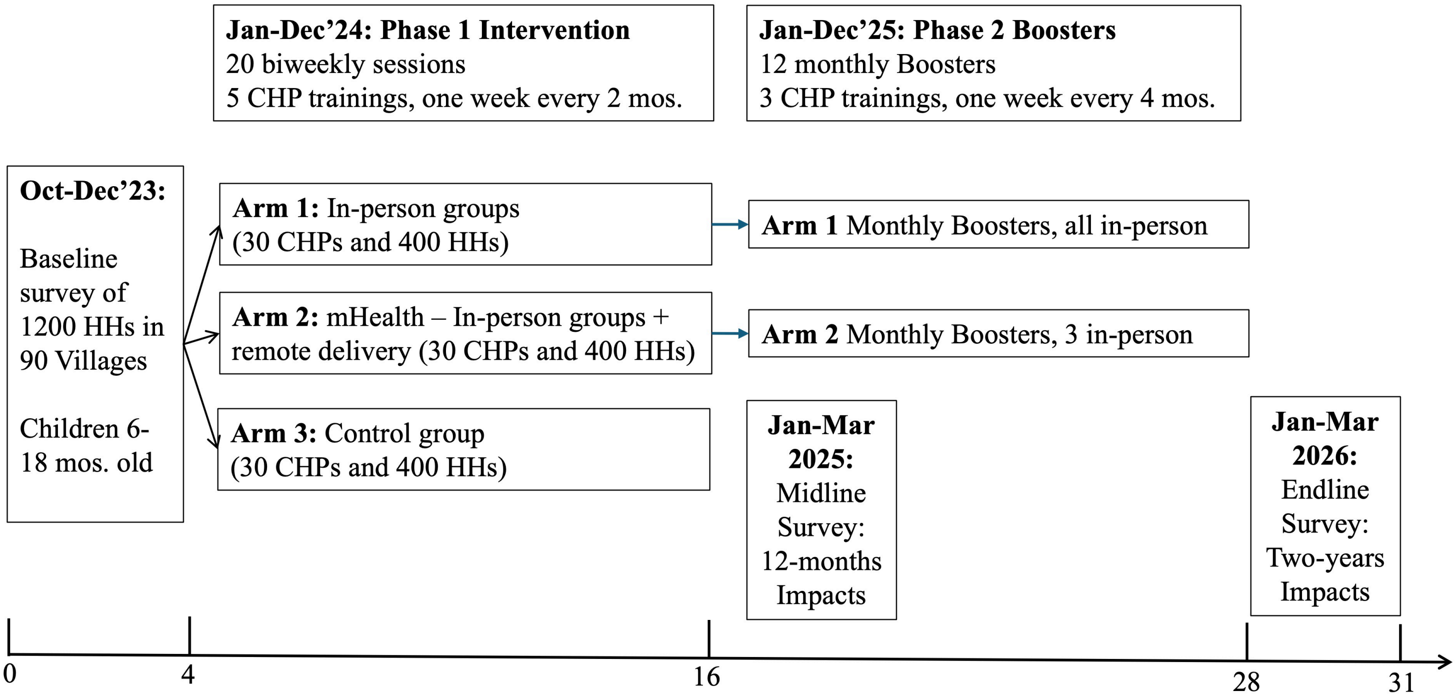

**Figure 1**: Msingi Bora mHealth Design and Timeline

### Figure 1

See image above for figure legend.

**Figure 2: Curriculum Design and Session Contents Arms 1 and 2**

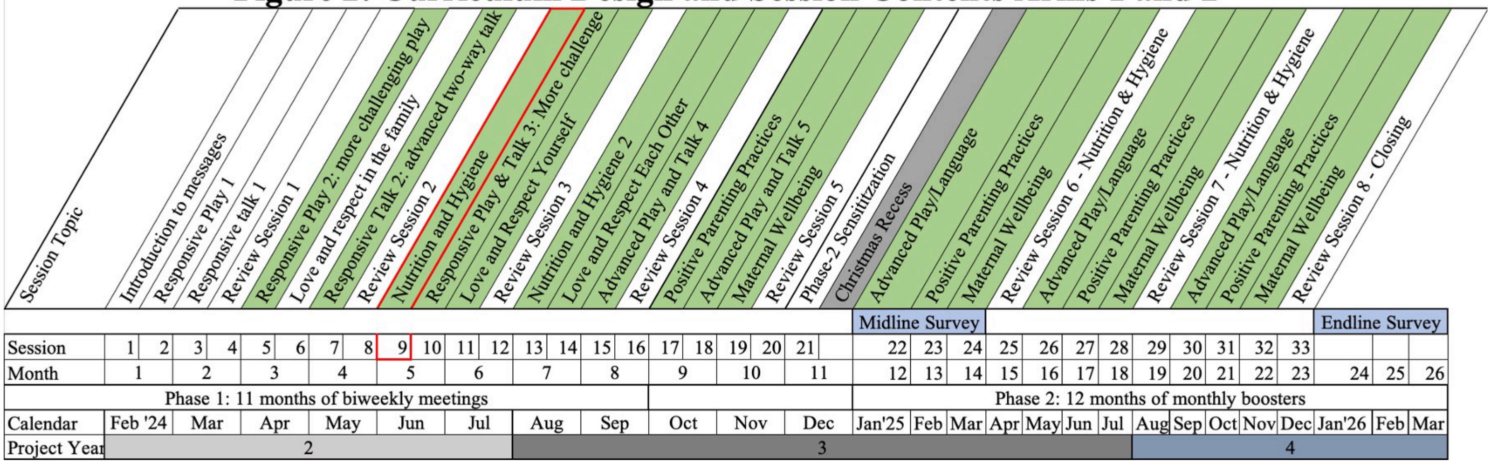

### Figure 2

See image above for figure legend.
